# Comparative Assessment of Five Decalcification Methods for Human Dental Tissue: An Ex Vivo Laboratory Study

**DOI:** 10.1155/ijod/9951057

**Published:** 2026-08-18

**Authors:** Anna Faruzelová, Jana Vaculová, Lucie Vrlíková, Jan Štembírek, Jakub Buday, Valeria Skopelidou, Marcela Buchtová, Pavel Hurník, Pavel Pořízka, Jozef Kaiser

**Affiliations:** ^1^ CEITEC Brno University of Technology, Purkyňova 656/123, 61200 Brno, Czech Republic; ^2^ Institute of Molecular and Clinical Pathology and Medical Genetics, University Hospital Ostrava, 17. listopadu 1790/5, 708 52 Ostrava-Poruba, Czech Republic, fno.cz; ^3^ Institute of Molecular and Clinical Pathology and Medical Genetics, Faculty of Medicine, University of Ostrava, Syllabova 19, 703 00, Ostrava, Czech Republic, osu.cz; ^4^ Laboratory of Molecular Morphogenesis, Institute of Animal Physiology and Genetics, Czech Academy of Sciences, Veveří 967/97, 602 00, Brno, Czech Republic, cas.cz; ^5^ Department of Oral and Maxillofacial Surgery, University Hospital Ostrava, 17. listopadu 1790/5, 708 52, Ostrava-Poruba, Czech Republic, fno.cz; ^6^ Faculty of Mechanical Engineering, Brno University of Technology, Technická 2896/2, 61669, Brno, Czech Republic, vutbr.cz; ^7^ Department of Experimental Biology, Masaryk University, Brno, Czech Republic, muni.cz

**Keywords:** demineralization, histological staining, laser-induced breakdown spectroscopy, teeth

## Abstract

**Background:**

Histological examination of human teeth is essential for the diagnosis of developmental anomalies, odontogenic lesions, and other dental pathologies. However it remains technically challenging because highly mineralized tissues require extensive processing before thin sections suitable for microscopic evaluation can be obtained. This study aimed to identify a decalcification protocol suitable for routine dental pathology by balancing rapid processing with high‐quality tissue preservation. To achieve this, we compared five commonly used decalcification protocols using complementary histological and spectroscopic analyses.

**Methods:**

Forty‐nine human teeth were decalcified using one of the five investigated agents: HNO_3_, HCOOH, DC1, Microdecfast, or Löwy’s solution. Decalcification speed, sectioning quality, and histological staining characteristics (H&E, PAS, Gömöri, and von Kossa) were evaluated. Laser‐induced breakdown spectroscopy (LIBS) was used for spectral analysis, 2D elemental mapping, and semi‐quantitative assessment of Ca, Mg, P, and C, enabling direct comparison of demineralization efficiency and spatial homogeneity across agents.

**Results:**

Decalcification efficiency differed markedly among the tested agents. HNO_3_ provided the fastest and most consistent tissue softening, whereas Löwy’s solution was the slowest and often failed to achieve complete decalcification. LIBS demonstrated that HNO_3_ and DC1 produced the most homogeneous depletion of Ca, Mg, and P, while Microdecfast and HCOOH resulted in less uniform mineral removal. All decalcifying agents depleted mineral elements beyond calcium, indicating that some protocols (HNO_3_ and DC1) may be unsuitable for studies requiring preservation of the native elemental composition. Less homogeneous demineralization was associated with harder tissues, inferior sectioning quality, and increased sample fragmentation.

**Conclusions:**

By integrating histology with LIBS analysis, this study demonstrates that decalcifying agents differ substantially in their decalcification speed, tissue preservation, and mineral removal profiles. Notably, 5% HNO_3_ emerged as the most efficient agent, enabling rapid decalcification with preserved staining quality and homogeneous mineral depletion. These findings provide practical guidance for diagnostic histopathology laboratories by facilitating faster processing of dental specimens while maintaining the tissue quality required for accurate microscopic diagnosis.

## 1. Introduction

Microscopic examination of dental tissues is an essential component of diagnostic pathology, supporting the diagnosis of developmental anomalies, mineralization disorders, odontogenic cysts and tumors, inflammatory periapical lesions, and traumatic or iatrogenic injuries affecting the teeth and surrounding tissues [1–9]. Accurate histopathological evaluation is therefore critical for appropriate clinical management. However, human teeth are among the most challenging specimens for histological processing because they contain highly mineralized tissues with distinct structural and mechanical properties, making routine sectioning impossible without prior decalcification. Consequently, the quality of microscopic evaluation depends largely on the efficiency of the decalcification protocol, which must sufficiently soften the specimen while preserving tissue morphology, staining characteristics, and antigenicity.

Chemical decalcification remains the most widely used method for preparing mineralized tissues for histological examination in routine diagnostic laboratories [10–14]. Although decalcification protocols for bone specimens are well established, processing human teeth presents additional challenges owing to the presence of enamel and the heterogeneous composition of dental tissues [11, 12, 15]. The effectiveness of decalcification depends on multiple factors, including specimen size, degree of mineralization, composition, concentration, and pH of the decalcifying solution [16, 17]. Careful control of the decalcification process is essential because insufficient decalcification hinders sectioning, whereas prolonged exposure may damage tissue architecture, impair staining quality, and compromise subsequent microscopic evaluation [2, 16–18].

Currently available decalcifying agents differ primarily in the balance between processing speed and preservation of tissue integrity. Strong mineral acids, such as nitric or hydrochloric acid, provide rapid decalcification but may adversely affect tissue morphology if exposure is prolonged [15, 19, 20]. In contrast, weaker acids and chelating agents, including ethylenediaminetetraacetic acid (EDTA) and nitrilotriacetic acid, generally better preserve microscopic architecture but require substantially longer processing times [1, 2, 19, 21–24]. Various approaches, such as increasing temperature, agitation, or regular replacement of the decalcifying solution, have been proposed to accelerate the process, although these modifications may themselves influence tissue preservation [1, 25–29]. Despite numerous comparative studies evaluating selected decalcifying agents [1, 2, 15, 19–22, 25, 26, 30–36], a comprehensive assessment of a broad range of routinely used decalcification protocols, integrating both histological quality and elemental changes in dental tissues, remains unavailable.

While numerous studies have evaluated the histological performance of different decalcifying agents, their effects on the elemental composition of dental tissues remain largely unexplored. Laser‐induced breakdown spectroscopy (LIBS) is well‐suited to address this knowledge gap because it enables rapid, spatially resolved, multielemental analysis directly from biological specimens, providing semi‐quantitative maps of elemental distribution (bioimaging) [37–41]. LIBS has been widely applied to characterize the elemental composition of mineralized tissues, including teeth, bone, and other calcified materials [42–44]. However, to the best of our knowledge, it has not previously been used to evaluate changes in mineral composition induced by different decalcification protocols. Therefore, integrating LIBS with conventional histological assessment offers a unique opportunity to compare decalcifying agents not only with respect to tissue preservation but also according to their effects on the spatial distribution and depletion of mineral elements.

Despite continuous improvements in decalcification techniques, the prolonged processing time required for human teeth remains a major limitation in routine diagnostic practice, with some protocols requiring several weeks or even months to complete [22, 24]. Conversely, more aggressive decalcifying agents can substantially accelerate processing but may compromise tissue morphology and staining quality, thereby limiting diagnostic reliability. Identifying a protocol that provides an optimal balance between rapid decalcification and preservation of tissue integrity is therefore essential for efficient histopathological evaluation of dental specimens. To address this need, we systematically compared five commonly used decalcifying agents by evaluating (i) decalcification time, (ii) the histological quality of the processed tissues, and (iii) changes in mineral composition using LIBS. We hypothesized that the investigated agents would differ not only in processing efficiency and preservation of tissue morphology but also in the extent and spatial distribution of mineral depletion.

## 2. Materials and Methods

### 2.1. Samples Collection

Human teeth were extracted during the treatment and processed into decalcification solutions. One tooth was not decalcified and was left as a reference for LIBS. This study exclusively included teeth with normal morphology, devoid of macroscopic signs of evident pathologies such as integrity damage or carious changes. All samples were measured and weighed before and after the decalcification process. Fixation in 10% neutral buffered formalin (Histofor BFS, Emedis, Červený Kostelec, Czech Republic) was performed for at least 24 h.

The study was conducted in accordance with the Declaration of Helsinki. The institutional Ethics Committee of University Hospital Ostrava reviewed the project and granted ethical approval (Number 503/2016). All teeth were extracted as part of standard dental treatment, and patients provided informed consent for the use of the biological material for research purposes.

### 2.2. Decalcification of Teeth

The tooth samples were separated into groups and subjected to different decalcification agents as follows: 5% nitric acid (HNO_3_) (Sigald, Prague, Czech Republic, 10 samples at room temperature), 8% formic acid (HCOOH) (Lach‐Ner, Neratovice, Czech Republic, 10 samples at room temperature), DC1 (Labonord, Templemars, France, four samples at room temperature), Microdecfast (Diapath, Martinengo, Italy, 10 samples at room temperature and five samples at 58°C), and Löwy’s solution (Penta, Prague, Czech Republic, 10 samples at room temperature).

HNO_3_ is known as a strong acid decalcifier, and HCOOH belongs to the group of weaker acids. Other used solutions are commercially produced where DC1 solution contains formic acid and formaldehyde, Microdecfast is a mixture of formic acid and hydrochloric acid, and Löwy’s solution is an agent containing, in addition to formaldehyde, acetic acid, and mercuric chloride. In this study, we excluded chelating agents from consideration due to their slow pace of processing, which poses complications in clinical practice (despite their benefit of being non‐damaging to the tissue). The tubes with immersed teeth were shaken regularly, with individual solutions being replaced every 3 days. In the case of microscopically visible precipitated salts, the solution was replaced more frequently. The decalcification status was regularly monitored (daily for the first 20 days and every 2–3 days thereafter) with a needle puncture method (a needle was forced into the crown of the tooth and if passing through the entire tooth, decalcification was considered complete). This approach, widely utilized in routine histopathological practice, is a common way of controlling the decalcification endpoint [26, 45]. If the tooth exhibited adequate softening, the decalcification process was halted by immersion in a rinsing solution. Further treatment was only administered once the tooth was deemed sufficiently decalcified. Additionally, a reduction in tooth weight served as another monitored indicator of the decalcification process.

### 2.3. Tissue Processing for Histological Analysis

Following the decalcification process, the teeth underwent a second round of measurements and weight assessment. Larger teeth were bisected along the longitudinal axis. After washing under running tap water for 15 min, the samples were processed using the standard protocol in a VIP6 tissue processor. The specimens underwent dehydration in alcohols, clarification in xylenes, and wax saturation under vacuum conditions. Thus prepared samples were embedded in paraffin (Bawax Plus, Bamed, Litvinovice, Czech Republic, 54–56°C), cut into 5 µm‐thick sections, and stained with Hematoxylin–Eosin (Lach‐Ner, Neratovice, Czech Republic). PAS staining (Penta, Prague, Czech Republic) and Gömöri’s silver impregnation staining (Penta, Prague, Czech Republic) were used to visualize the basement membrane and highlight reticulum fibers. Von Kossa staining was used to evaluate the presence of calcium salts. When the sectioning proved challenging and macroscopic evidence suggested an inadequate decalcification of the FFPE‐processed tooth (notably, white and hard areas), surface decalcification was applied. This step involved immersion of the entire FFPE block in a suitable decalcification solution for a brief period (minutes to tens of minutes). Surface decalcification; however, permits the generation of only a limited number of sections and; therefore, requires repetitions until the desired quantity of sections is achieved. Microscopic evaluation of the quality of staining and morphology of the resulting tooth samples was performed by a pathologist using a light microscope. Parameters evaluated microscopically included the ease of sectioning and its quality (crumbling and cracks), nuclear and collagen staining quality of both soft (pulp) and hard parts of the tooth sample, the sample integrity, degree of decalcification, and the overall appearance of the resulting histopathological specimen. A scale from 1 to 3 was used for the microscopic evaluation (1: weak, 2: suitable, and 3: excellent).

### 2.4. Spectroscopic Analysis

In addition to microscopic evaluation, spatially resolved elemental imaging using LIBS was conducted to provide further insight into the alterations in the composition of the dental tissue. For spectroscopic examination, 2–4 randomly selected samples from each decalcifying agent group were analyzed. In total, 19 samples, including a non‐decalcified tooth, were analyzed using the FireFly LIBS system (Lightigo, Brno, Czech Republic) equipped with a Nd:YAG laser (266 nm, 8 ns, 50 Hz, and 10 mJ). The laser beam was focused on the sample surface into a spot size of 30 µm. Plasma emission was captured and transferred through a 400 µm optical fiber to a Czerny‐Turner spectrometer with a spectral range of 240–410 nm, a 25 µm entrance slit, and an embedded CMOS detector. All measurements were performed under an argon purge (9 L min^−1^) for profound plasma formation and signal intensity enhancement. Samples were analyzed by mapping the sample surface with a step size of 30 µm in both the *X* and *Y* axes. The analyzed sample movement between laser pulses was ensured using a computer‐controlled motorized stage. The map sizes varied depending on sample sizes, ranging from 89,100 to 324,800 spectra (Table S1 in the Supporting Information), leading to analysis times of approximately 30–110 min.

### 2.5. Spectroscopic Data Analysis

Spectral lines (Table 1) of several biogenic elements, such as calcium, magnesium, phosphorus, and carbon, were analyzed based on the obtained spectra. All measured spectra were processed and plotted in Lightigo ImageLab software (Epina Software Labs, Retz, Austria) or in OriginPro 2024 (OriginLab Corporation, Northampton, MA, USA). Subsequently, 2D distributional maps for the selected elements were generated from the processed spectra in ImageLab software. Only a few spectral lines (highlighted in bold in Table 1) were selected for the generation of elemental maps. To mitigate matrix effects, the standardization of selected elemental lines was performed using C I 247.86 nm as carbon is present in both the tooth and the surrounding paraffin. Standardization was performed in each map pixel by the spectral line ratio, where the selected biogenic element spectral line intensity (Ca, Mg, and P) was divided by the carbon line intensity. This approach enabled a more reliable semiquantitative comparison between samples from all agents.

**Table 1:** List of analyzed spectral lines and their properties [46].

Spectral line	*λ* (nm)	*A*_ki_ (10^8^ s^−1^)	*E*_*i*_ (eV)	*E*_*k*_ (eV)
**C I**	**247.86**	**2.80**	**2.68**	**7.69**
**P I**	**253.56**	**0.95**	**2.32**	**7.21**
P I	255.33	0.71	2.32	7.18
**Mg II**	**279.55**	**2.60**	**0.00**	**4.43**
Mg II	280.27	2.57	0.00	4.42
Mg I	285.21	4.91	0.00	4.35
Ca II	315.89	3.10	3.12	7.05
Ca II	317.93	3.60	3.15	7.05
Ca I	364.44	0.35	1.90	5.30
Ca II	370.60	0.88	3.12	6.47
Ca II	373.69	1.70	3.15	6.47
Ca II	393.37	1.47	0.00	3.15
**Ca II**	**396.85**	**1.40**	**0.00**	**3.12**

*Note:* Bold values were used for the generation of elemental maps in figures.

**Figure 1:**
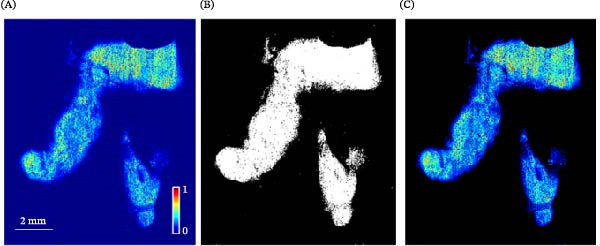
Process of data filtering preceding data segmentation. (A) Measured elemental map. (B) Mask with selected tooth spectra with intensities three times higher than the standard deviation of the background. (C) Final elemental map, after applying the mask on the measured map.

Moreover, the semi‐quantitative comparison of decalcifying agents involved calculating the relative average intensities of selected elemental spectral lines for each sample. Only spectra corresponding to the teeth, excluding epoxy, were considered. Specifically, spectra with intensities of the selected spectral lines exceeding three times the standard deviation of the background signal were considered signals (Figure 1). These spectra were afterward individually standardized to the C I 247.86 nm spectral line. Following this, the average intensity of the chosen spectral line for the entire tooth was computed by summing up all spectral line intensities for the particular tooth and dividing the sum by the number of spectra. The resultant average intensities for each decalcifying agent provided semiquantitative information for their comparative analysis. This process is visualized in Figure 1.

## 3. Results

### 3.1. Microdecfast and HNO_3_ Demonstrated the Fastest Decalcification of Human Teeth

**Figure 2:**
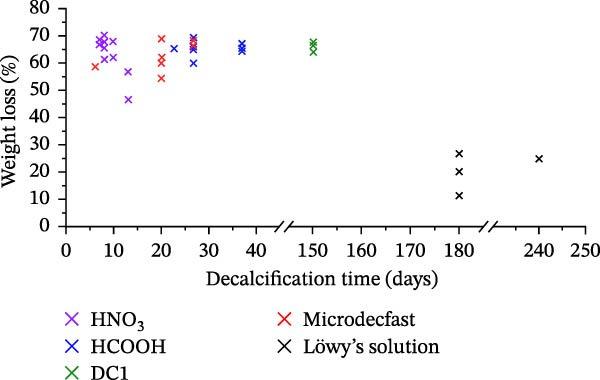
Weight loss as a function of the decalcification period for individual agents.

First, we evaluated the speed of tooth decalcification (i.e., time to complete decalcification) by several solutions commonly used for this purpose, namely, HNO_3_, HCOOH, DC1, Microdecfast, and Löwy’s solution. Unfortunately, the batch of five teeth subjected to decalcification in Microdecfast at 58°C disintegrated and, as such, were removed from further analyses, leaving 44 teeth (10 per agent, with the exception of DC1 with four teeth only). The time needed for the complete decalcification of dental tissue varied considerably among the tested solutions (Figure 2, Table 2, Tables S2–S6 in the Supporting Information; Kruskal–Wallis test *p* < 0.001). Four agents, namely, HNO_3_, HCOOH, DC1, and Microdecfast decalcified the samples to a similar degree, resulting in approximately 65% weight loss. Still, they differed in the decalcification speed. While the use of HNO_3_ resulted in the fastest decalcification of dental tissue (median 182 h, min–max 168–312 h), which was significantly lower than all other agents, including Microdecfast (median 562 h; 146–642 h, *p* = 0.002) and HCOOH (median 765 h, 542–888 h, *p* = 0.001). Microdecfast was also statistically significantly slower acting than HCOOH (*p* = 0.03). The decalcification process was much slower for the two remaining agents—DC1 and Löwy’s solution. In these cases, the procedure proved to be highly time‐consuming (5–8 months), and most samples (six out of 10) immersed in Löwy’s solution could not be adequately processed even after 20 months. For this reason, these samples were not included in the statistical comparison. Additionally, a clear inefficiency of Löwy’s solution for tissue decalcification was proved by a lower weight loss (~22%) compared to the rest of the used agents.

**Table 2:** Numerical descriptors of decalcification efficiency of individual agents.

Decalcifying agent	HNO_3_	HCOOH	DC1	Microdecfast	Löwy’s solution
Decalcification duration	8 ^∗^ (7–13) days	32 ^∗^ (23–37) days	5 months ^∗^	23 ^∗^ (6–27) days	6+ months^NA^
Loss of weight (%)	66.5 (46.6–70.3)	65.4 (60.0–67.0)	65.5 (64.2–66.7)	66.4 (54.5–69.1)	22.6^NA, a^ (11.0–26.8)

^a^Four teeth were evaluated, passing the needle test.

^∗^Values with asterisks are mutually all statistically significantly different.

^NA^Not included in statistical evaluation due to incomplete decalcification of all teeth.

### 3.2. Decalcification Agents Influenced the Quality of Dental Tissue to Varying Degrees

**Figure 3:**
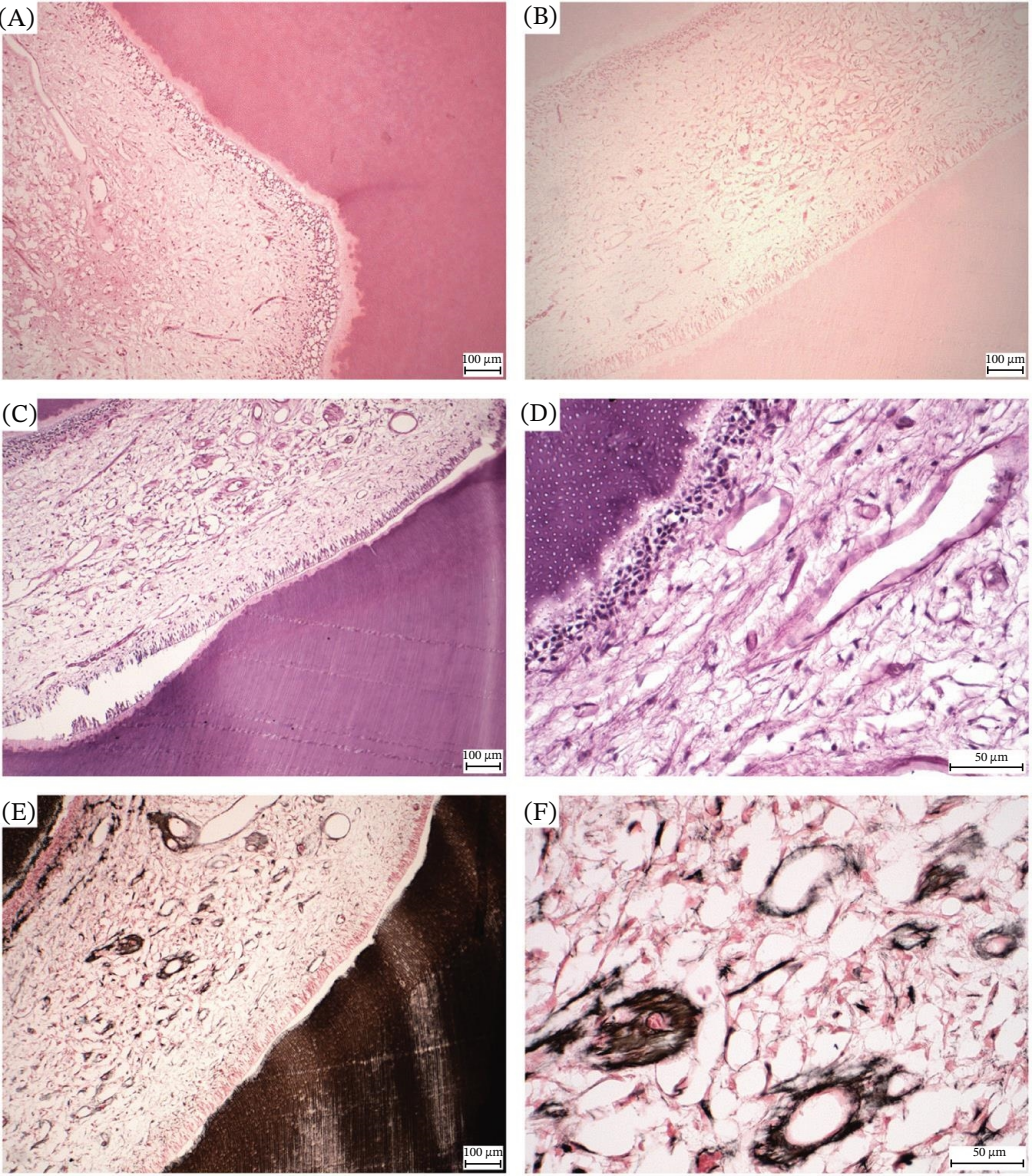
Outcomes of decalcification of human dental tissue with HNO_3_ solution—evaluation of histological and staining quality. (A) Hematoxylin–Eosin staining, magnification × 100, (B) Von Kossa staining, magnification × 100, (C, D) PAS staining, × 100 and × 400 magnification, and (E, F) Gömöri’s silver impregnation, × 100 and × 400 magnification.

**Figure 4:**
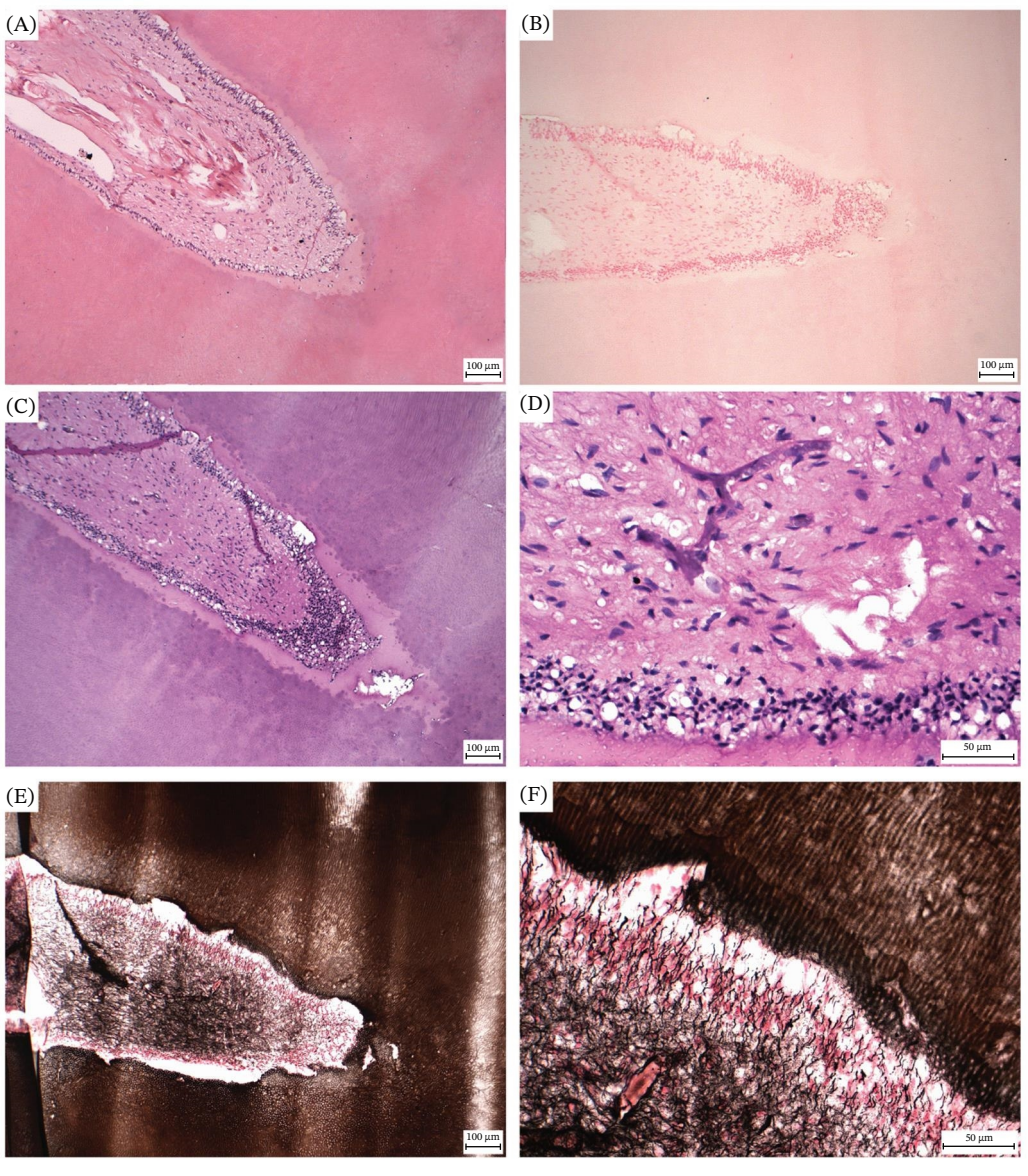
Outcomes of decalcification of human dental tissue with HCOOH—evaluation of histological and staining quality. (A) Hematoxylin–Eosin staining, magnification × 100, (B) Von Kossa staining, magnification × 100, (C, D) PAS staining, × 100 and × 400 magnification, and (E, F) Gömöri’s silver impregnation, × 100 and × 400 magnification.

**Figure 5:**
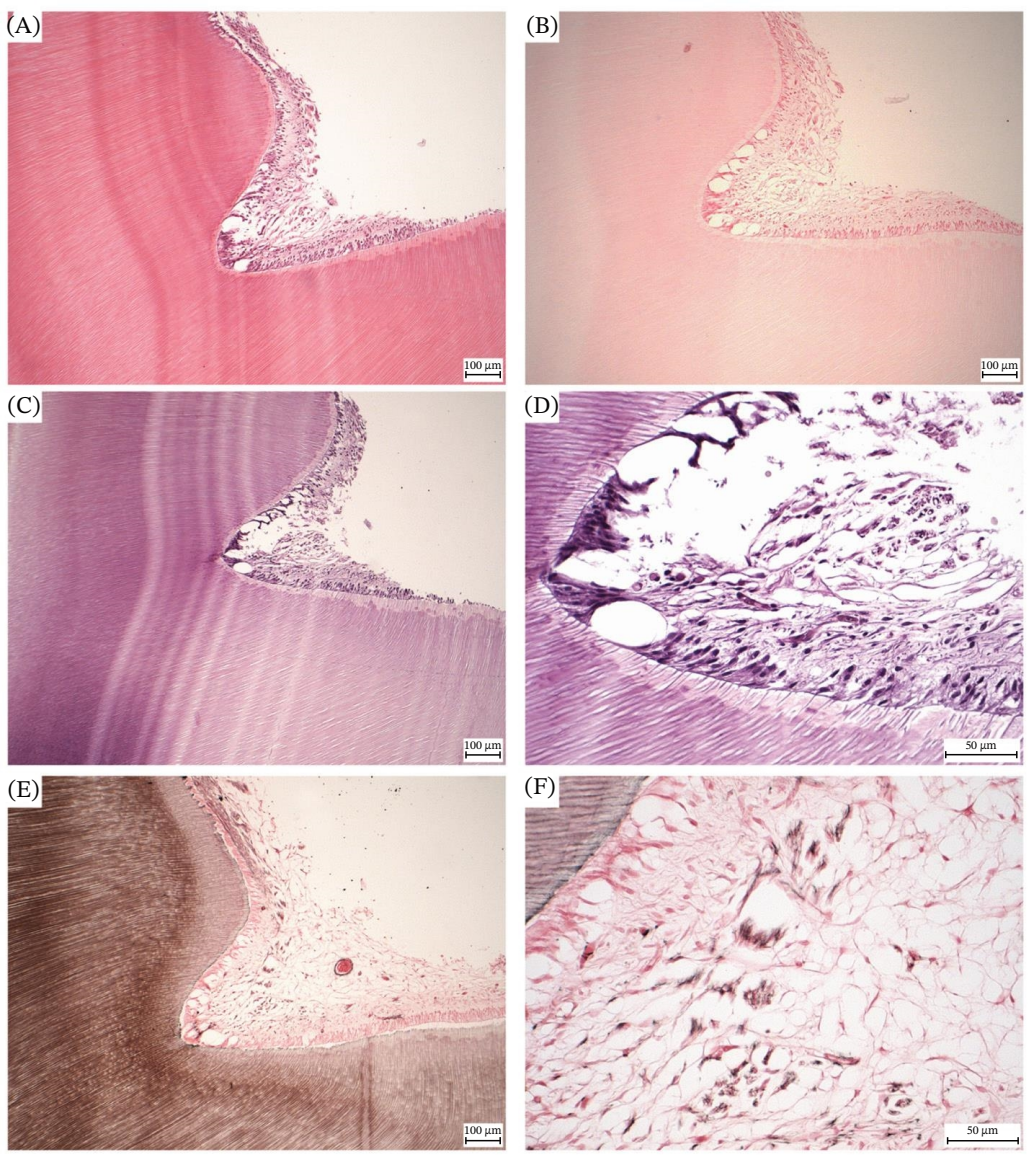
Results of decalcification of human dental tissue with DC1 solution—evaluation of histological and staining quality. (A) Hematoxylin–Eosin staining, magnification × 100, (B) Von Kossa staining, magnification × 100, (C, D) PAS staining, × 100 and × 400 magnification, and (E, F) Gömöri’s silver impregnation, × 100 and × 400 magnification.

**Figure 6:**
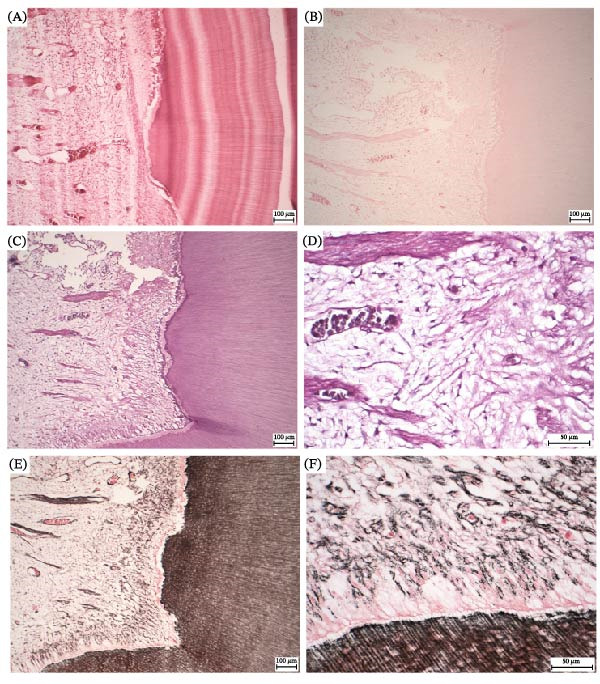
Outcomes of decalcification of human dental tissue with Microdecfast—evaluation of histological and staining quality. (A) Hematoxylin–Eosin staining, magnification × 100, (B) Von Kossa staining, magnification × 100, (C, D) PAS staining, × 100 and × 400 magnification, and (E, F) Gömöri’s silver impregnation, × 100 and × 400 magnification.

**Figure 7:**
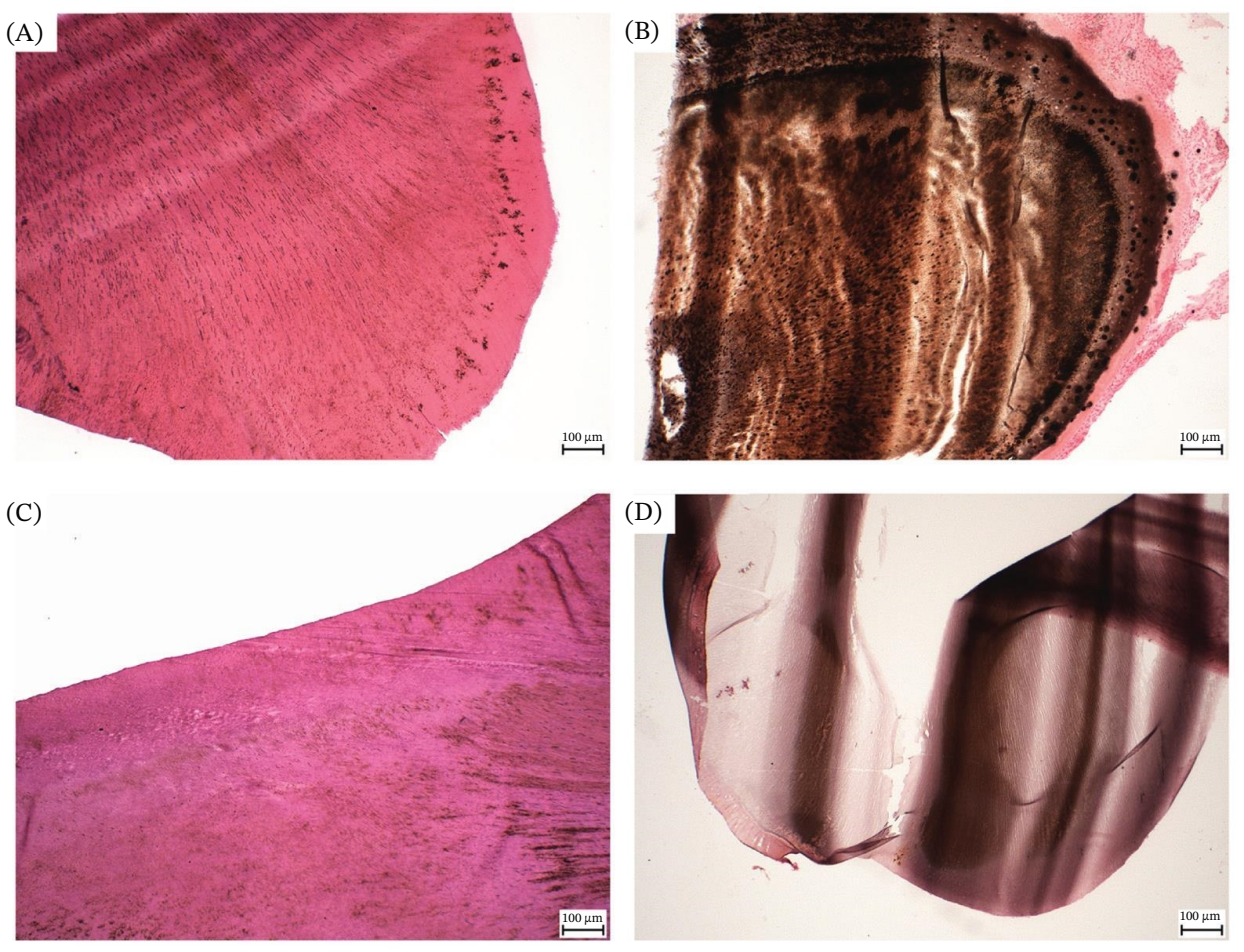
Outcomes of decalcification of human dental tissue with Löwy’s solution—evaluation of histological and staining quality. (A) Hematoxylin–Eosin staining, magnification 100x, (B) Von Kossa staining, magnification × 100, (C) PAS staining, × 100 magnification, and (D) Gömöri’s silver impregnation, × 100 magnification.

In addition to the needle puncture method, the quality of tooth decalcification was also evaluated from other perspectives (Table 3)—macroscopically (during the slicing on the microtome) and microscopically (Figures 3–7). Von Kossa staining was employed for microscopic assessment of the quality of decalcification. All decalcifying agents, with the exception of Löwy’s solution, exhibited satisfactory decalcification of the teeth, as evidenced by the absence of calcium salts in the sections.

**Table 3:** Results representing the quality of histological processing and histochemical staining (always two teeth out of the set of teeth decalcified using the same solution were selected for evaluation).

Agent	Sample	Sectioning ease	Section quality	H&E nuclear staining quality	Pulpal morphology (sample integrity)	PAS staining quality	Gömöri staining quality	Von Kossa (degree of decalcification)	Decalcification time (days)
HNO_3_	N1	2	2	1	2	2	2	3	13
	N10	2	2	2	1	3	3	3	5.5
HCOOH	F6	1	1	3	2	3	2	3	37
	F10	1	1	3	2	2	3	3	23
DC1	D3	2	1	2	1	2	1	3	152
	D5	2	1	2	2	2	2	3	152
Microdecfast	M3	2	3	1	2	2	3	3	6.1
	M10	2	3	1	1	2	3	3	27
Löwy’s solution	L1	1	1	2	N	3	3	1	183
	L4	1	1	2	N	N	N	N	183

*Note:* Evaluation scale from 1 to 3 (1: weak, 2: suitable, and 3: excellent), H&E, Hematoxylin–Eosin staining; N, not possible.

#### 3.2.1. Assessment of Tissue Decalcification During Microtome Processing

Slicing teeth decalcified with HCOOH (Figure 4A–F) and Löwy’s solution (Figure 7A–D) proved to be the most challenging. On the other hand, slicing of samples decalcified in HNO_3_, DC1, and Microdecfast solutions (Figures 3A–F, 5A–F, and 6A–F) was relatively easy (i.e., neither the tooth nor the sections were prone to crumbling during sectioning, or the tissue sections were free of breaks). Still, none of the decalcified teeth were sectioned as effortlessly as that in normal soft tissues.

#### 3.2.2. Evaluation of Histological Sections Quality

The sections from teeth decalcified in Microdecfast were the most representative (highest quality, smooth, and even), although their slicing was more difficult than the slicing of teeth decalcified in HNO_3_. The quality of HNO_3_‐decalcified teeth ranked second best. Unfortunately, producing high‐quality sections from specimens decalcified in HCOOH, DC1, and Löwy’s solution proved challenging (even impossible in the case of Löwy’s solution) as the samples were excessively hard and prone to crumbling.

#### 3.2.3. Examination of Pulpal Morphology, Including Sample Integrity

Full pulp cohesion was not evident in any of the examined teeth. Every decalcifying solution resulted in either partial or complete separation of the pulp from the wall of the pulp cavity (Table 3 and Figures 3–7).

#### 3.2.4. Nuclear Staining With Hematoxylin

The highest quality of nuclear staining in H&E was observed in teeth decalcified with HCOOH, while the least favorable results were observed in samples processed with Microdecfast and some of those decalcified by HNO_3_ (Table 3 and Figures 3–7).

#### 3.2.5. PAS Staining

Basement membranes were evident in all samples; excellent staining was; however, observed only in some samples decalcified using HCOOH, HNO_3_, and Löwy’s solution (Table 3 and Figures 3–7).

#### 3.2.6. Gömöri’s Impregnation Method

Reticular fibers were identifiable in all examined samples, with the most favorable outcomes observed in those treated with Microdecfast, although HNO_3_ and HCOOH also yielded satisfactory results (Table 3 and Figures 3–7).

### 3.3. LIBS Analysis Uncovered HNO_3_ and DC1 as the Most Effective Demineralizing Agents

**Figure 8:**
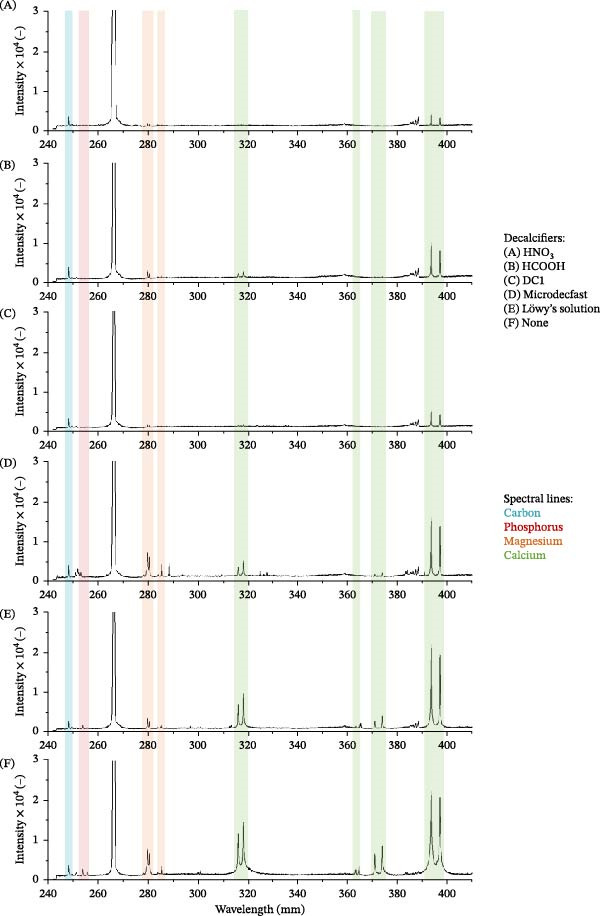
Mean spectra of teeth demineralized in individual agents. (A) HNO_3_, (B) HCOOH, (C) DC1, (D) Microdecfast, (E) Löwy’s solution, and (F) nondecalcified. Color‐coded lines of carbon, phosphorus, magnesium, and calcium are included. The 266 nm line corresponds to the UV ablation laser.

A noticeable distinction among all employed decalcifying agents is evident in the spectral analysis (Figure 8A–F). The spectra of teeth decalcified using Löwy’s solution displayed intensities comparable to the control (nondecalcified) tooth spectrum, indicating its limited decalcifying efficiency. Spectral lines of all biogenic elements were discernible, with phosphorus being specifically detected only in this sample group (Figure 8E). This is not surprising given that the decalcification was incomplete in this medium (and, therefore, calcium phosphate was obviously not disintegrated). Microdecfast and HCOOH were more efficient than Löwy’s solution. Although they demonstrated comparable results, the Microdecfast spectrum exhibited higher intensities of Mg lines (Figure 8D), potentially indicating a somewhat lower efficiency in removing other minerals from the dental tissue. HNO_3_ and DC1 were the most effective decalcifying agents among the analyzed solutions (note; however, the very long time needed for DC1 decalcification). Both exhibited extremely low or no signals for all analyzed elements, with only ionic lines of magnesium (Mg II 279.55 nm and Mg II 280.27 nm) and calcium (Ca II 393.37 nm and Ca II 396.85 nm) being detected.

**Figure 9:**
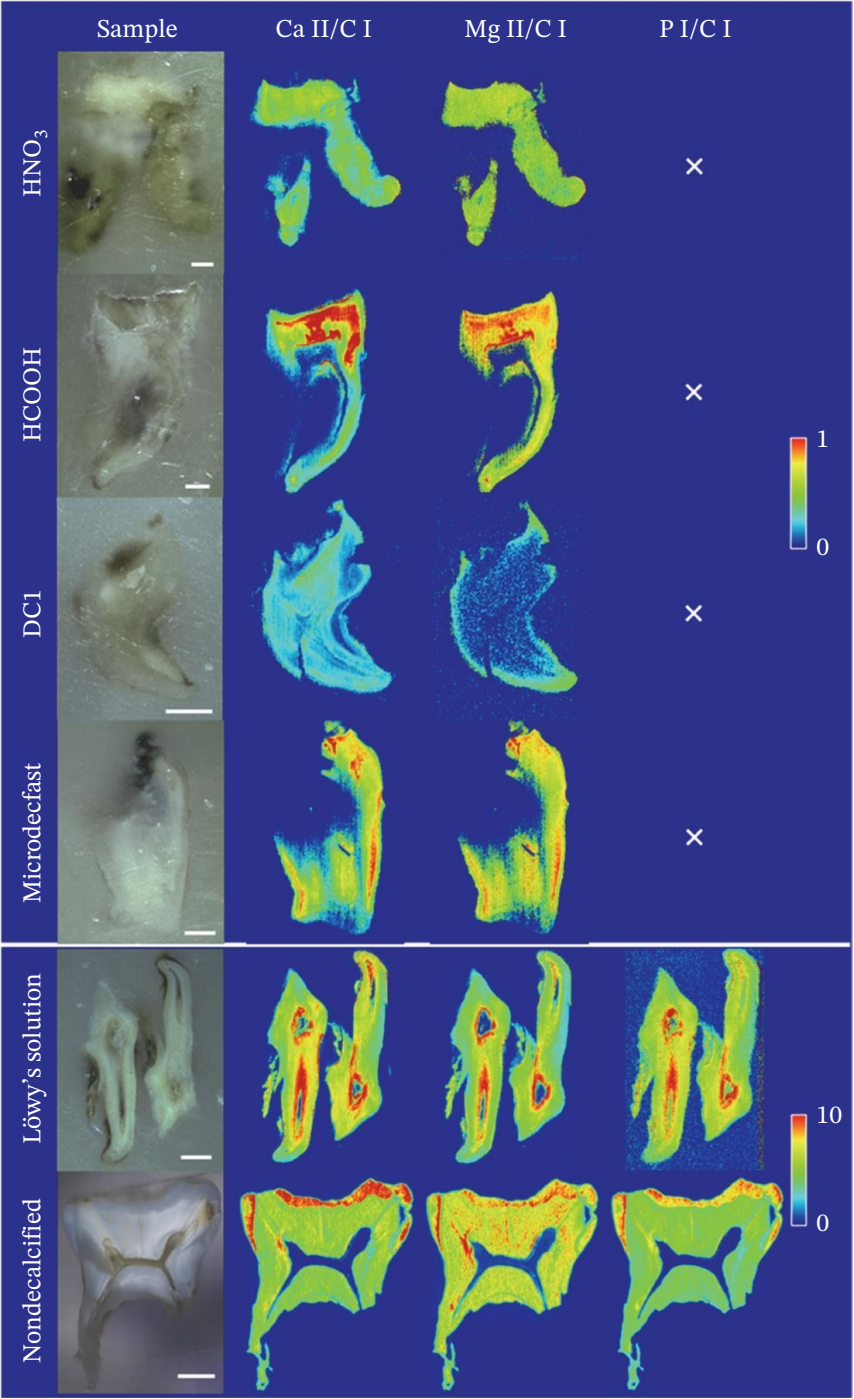
Distributional maps of detected biogenic elements. The spectral lines of Ca II 396.85 nm, Mg II 279.55 nm, and P I 253.56 nm were standardized to C I 247.86 nm for all samples and decalcification agents. Note that the nondecalcified and Löwy’s solution samples’ color scales are 10 times higher than those of the other decalcifiers due to high intensities (differences in color scales depicted by the white line across the figure). Scale bars are 2 mm (white lines in the bottom left corner of the sample photographs).

For a more comprehensive illustration of the influence of each decalcifying agent, 2D elemental maps were generated to visualize their spatial distribution in addition to their decalcification efficiency. Below, maps of Ca II 396.85 nm, Mg II 279.55 nm, and P I 253.56 nm after the standardization by C I 247.86 nm (indicated by the labeled ratios—Ca II/C I, Mg II/C I, and P I/C I) for one representative sample from each decalcifying agent are presented to allow the comparison (Figure 9). As mentioned above, standardization to the carbon line was used to mitigate matrix effects, thus enabling the agents’ mutual comparison.

However, even after standardization, the alignment of the color scales of the nondecalcified tooth and Löwy’s solution samples with those of the other agents was still challenging. Given the considerably stronger spectral signals in the nondecalcified samples and those treated in Löwy’s solution, the color scales for their maps had to be adjusted from 0*–*1 to 0*–*10 due to image saturation. This adjustment is crucial to consider when comparing maps across different agent groups, further highlighting the limited decalcification efficiency of Löwy’s solution.

More consistent outcomes were achieved in the samples from the remaining agents, primarily indicated by the unsuccessful detection of phosphorus (white crosses in Figure 9) due to the spectral signal falling below the system’s detection limits. Nonetheless, minor distinctions were noted. For DC1 and HNO_3_ agents, decalcification exhibited greater uniformity across the entire sample. In contrast, the use of Microdecfast and HCOOH agents led to less homogeneous decalcification, as evidenced by higher‐intensity areas in the elemental maps for both calcium and magnesium.

To improve visualization, the color scale values in the figures were standardized. This normalization was conducted individually for each element, allowing for the comparison of maps for each element across all analyzed agent groups.

### 3.4. Individual Elements are Removed With Varying Efficiency by Decalcification Agents

**Figure 10:**
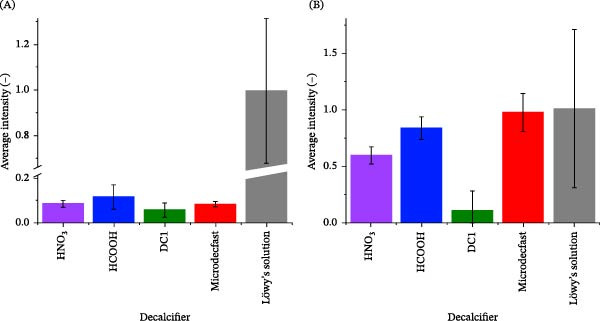
Average relative intensities of (A) Ca II 396.85 nm and (B) Mg II 279.55 nm lines for each decalcifying agent group.

To obtain a semiquantitative analysis and comparison of the decalcification quality between the agents, data segmentation of the generated elemental maps was performed (Figure 1). After the spectra filtration and standardization to the C I 247 nm line, the average relative intensity of calcium Ca II 396.85 nm and magnesium Mg II 279.55 nm lines was calculated and visualized for each agent (Figure 9). Figure 10 presents the average values of the intensities from LIBS analysis for all samples prepared using the corresponding agents. In the case of calcium (Figure 10A), a 10‐times higher intensity was obtained in samples acquired using Löwy’s solution, which necessitated rescaling of the vertical axis.

The results highlight the effectiveness of HCOOH and Microdecfast agents and the superior efficiency of HNO_3_ and DC1 agents. However, the level of magnesium (Figure 10B) displayed a distinct trend compared to the imaging analysis. Microdecfast exhibited a high intensity, comparable with Löwy’s solution, with a difference only in the degree of magnesium removal defined by a lower standard deviation. Following Microdecfast and Löwy’s solution, HCOOH was the next most efficient agent for magnesium removal. This implies that the decalcification process removes not only calcium salts but also other elements, albeit with varying efficiency. This should; therefore, be considered when choosing a decalcifying agent.

## 4. Discussion

Our study has revealed that the microscopic anatomy of the dental tissue undergoes fundamental changes based on the selected decalcification method. The use of strong acids or prolonged decalcification times is generally considered to increase the risk of tissue damage, disintegration, and the inability to perform valid histological details. This can adversely affect the staining of basophilic components and result in tissue maceration [22, 47]. Conversely, incomplete decalcification poses challenges in preparing high‐quality thin sections. In such instances, the surface decalcification of the block can be effectively employed just before microtome cutting [18, 24, 29].

The challenge of tissue sectioning is influenced by various factors. Essential considerations include the degree of fixation, tooth decalcification, effective tissue processing, and saturation with high‐quality, suitable paraffin [24, 36, 46, 48]. Additionally, the cutting technique, microtome type, the expertise of the lab technician, and the angle and type of microtome blades play crucial roles in determining the section’s quality. Obtaining intact and flat sections was found to be challenging for almost all teeth. Some sections crumbled and parts of the teeth peeled out of the block. The sections frequently appeared torn, wrinkled, incomplete, and exhibited uneven thickness. These factors could potentially impact the quality of staining, leading to occasional patchiness due to the nonuniformity of the sections.

Previous comparative studies primarily evaluated processing time together with preservation of tissue morphology [2, 23, 25–27, 30]. Our study extends these observations by incorporating spatially resolved elemental analysis, enabling direct comparison between microscopic tissue quality and mineral depletion. According to the results of Dey and Bancroft [19, 20], EDTA, HNO_3,_ and HCOOH are the most commonly used decalcifying solutions. Earlier studies consistently reported that EDTA provides excellent preservation of tissue morphology at the expense of prolonged processing, whereas HNO_3_ accelerates decalcification but may compromise histological quality [22, 32, 47, 48]. On the other hand, weak acids (e.g., HCOOH) were found to be slower but more gentle to the tissue [2, 31, 49, 50], which our findings confirmed only partially. As expected, HNO_3_ demonstrated the most favorable outcomes in terms of both speed and degree of decalcification, and, as mentioned above, the sectioning ease was highly satisfactory. Surprisingly; however, it also performed very well in preserving morphology and yielding samples highly suitable for staining. We assume this is likely caused by the everyday verification of the process using the needle puncture test, which allowed the timely termination of HNO_3_ action as soon as the decalcification was complete, before the acid could cause major damage to the softer tissues.

Although HCOOH and Microdecfast performed similarly well in tissue preservation, the processing times were higher, and from this perspective, their performance was inferior to that of HNO_3_. DC1 yielded both significantly longer decalcification times and inferior quality of the resulting sections. Löwy’s solution (consisting of formaldehyde, acetic acid, and mercuric chloride) demonstrated the slowest decalcification. Six teeth remained insufficiently processed even after 8 months, making paraffin embedding and standard slicing on a microtome impossible. Nevertheless, the procedure was suboptimal even for the four teeth that passed the needle puncture test as their decalcification was not complete (weight loss only ~23% compared to ~65% in the other solutions).

The endpoint of decalcification in this study was determined using the conventional needle puncture method, which is widely used in routine histopathological processing because of its simplicity and rapidity [26, 45]. However, this approach is minimally destructive and therefore unsuitable when the same specimen is intended for subsequent molecular, biochemical, or advanced imaging analyses. In such cases, nondestructive methods, such as radiography or microcomputed tomography, may provide more appropriate alternatives. Since each tooth in the present study was dedicated exclusively to histological evaluation, the endpoint assessment did not interfere with any downstream analyses or influence the study outcomes.

An important contribution of the present work is that LIBS independently confirmed the histological observations while simultaneously revealing differences that conventional microscopy alone could not detect. Specifically, elemental mapping demonstrated that agents producing similar microscopic appearances differed substantially in the extent and spatial homogeneity of mineral depletion. The DC1 decalcifier achieved slightly superior decalcification, although the consistency of results across the sample set was lower in this group. Löwy’s solution emerged as the least effective agent, with a level of minerals comparable to those in nondecalcified teeth. These findings were obtained from three LIBS data analyses—spectral, imaging, and data segmentation. Spectral and imaging analyses yielded closely aligned results, while data segmentation revealed a notable distinction in magnesium levels. In this context, the intensities of Löwy’s solution were similar to those of HNO_3_, but Microdecfast intensities exceeded those detected in teeth decalcified using other techniques. This overall finding aligns with our hypothesis that decalcifying agents remove, besides calcium salts, also other elements. This can vary across individual agents; however, it can significantly contribute to the quality of the generated samples. Therefore, the whole demineralization status should be taken into account, not just the removal of calcium.

One limitation of this study is the slightly unequal sample size among the experimental groups, as one intact tooth was reserved for baseline LIBS analysis of untreated mineralized tissue. Although this reduced the DC1 group to four specimens, the consistency of the histological and spectroscopic findings suggests that this minor imbalance did not influence the overall conclusions. However, given that the decalcification speed in this medium was so much longer than in HNO_3_, HCOOH, or Microdecfast, statistical significance was demonstrable despite the low number of teeth. Also, the fact that only two teeth out of the set were histopathologically evaluated is another limitation of this study. The use of the needle puncture test for determining the point of sufficient decalcification can also be considered a limitation as this method may miss local calcium deposits. Although other methods, such as X‐ray tissue examination or the use of ammonium oxalate, could be employed, the method we used is very simple and easy‐to‐ perform, which allowed us frequent checks and made it possible to stop decalcification before the agents cause damage to the soft tissues (for example, in the case of HNO_3_, for which we, likely thanks to this approach, received good quality of histological sections). On the other hand, the wide range of decalcification agents used in this head‐to‐head comparison and the complex analysis going beyond just the decalcification time, investigating the sectionability, stainability, tissue quality, and mineral composition, are a major strength of this study, allowing a comprehensive evaluation and drawing strong conclusions about the suitability of individual agents for the decalcification of teeth.

From a clinical perspective, optimizing decalcification protocols has the potential to reduce the turnaround time for the histopathological evaluation of extracted teeth and other mineralized dental specimens. Faster processing, while maintaining tissue morphology and staining quality, may facilitate earlier diagnosis of developmental anomalies, odontogenic lesions, and inflammatory diseases, thereby supporting timely clinical decision‐making. Within this clinical context, the present study focused specifically on parameters that directly influence routine histopathological diagnostics, namely, tissue preservation and mineral composition. The effects of decalcification on organic tissue constituents, such as proteins and lipids, were beyond the scope of this work and warrant further investigation, particularly for applications involving proteomics or lipidomics, where the preservation of biomolecular integrity is essential.

## 5. Conclusions

This study demonstrates that the evaluation of decalcification protocols should extend beyond processing time and conventional histological assessment. By integrating histology with LIBS, we identified previously unrecognized differences in the extent and spatial distribution of mineral depletion among routinely used decalcifying agents, providing a more comprehensive framework for selecting protocols for both diagnostic pathology and dental research. Among the five evaluated decalcifying agents, HNO_3_ showed the best overall performance, providing rapid decalcification while preserving tissue morphology, staining quality, and homogeneous mineral removal. Under our conditions, complete decalcification was achieved within approximately 2 weeks, supporting its suitability for routine histopathological processing of dental specimens. Microdecfast represented a reasonable alternative, whereas HCOOH, DC1, and Löwy’s solution were less suitable because of slower or less effective decalcification and reduced tissue quality.

Beyond identifying the most effective decalcification protocol, our findings demonstrate that different agents differentially affect the depletion of mineral elements in addition to calcium, including magnesium and phosphorus. These findings demonstrate that selecting an appropriate decalcification protocol is important not only for preserving tissue quality but also for improving the efficiency and reliability of histopathological diagnosis in routine dental pathology. In addition, awareness of decalcification‐induced changes in mineral composition may benefit future studies investigating the elemental characteristics of dental tissues.

Overall, this work provides practical guidance for optimizing decalcification protocols in diagnostic laboratories while establishing LIBS as a valuable complementary tool for evaluating the effects of decalcification on mineralized tissues.

## Author Contributions

**Anna Faruzelová and Jana Vaculová**: investigation, methodology, writing – original draft, visualization. **Lucie Vrlíková**: investigation, methodology, visualization. **Jan Štembírek**: resources (sample collection), funding acquisition. **Jakub Buday**: methodology, software, data curation. **Valeria Skopelidou**: investigation, visualization, writing – original draft. **Marcela Buchtová**: conceptualization, supervision, writing – original draft, funding acquisition. **Pavel Hurník**: conceptualization, supervision, writing – original draft, visualization, funding acquisition. **Pavel Pořízka**: conceptualization, supervision, software, funding acquisition. **Jozef Kaiser**: conceptualization, supervision, funding acquisition. All authors contributed to writing – review and editing of the final manuscript.

## Funding

This study was supported by the Ministry of Health of the Czech Republic (original analyses supported by grant NU20‐06‐00189 and revisions of the manuscript by grant AZV/NW24‐10‐00204) and by RVO/FNO 2022; by the project “Mechanical Engineering of Biological and Bio‐inspired Systems” (Grant CZ.02.01.01/00/22_008/0004634), funded through the Program Johannes Amos Comenius (Excellent Research) by Brno University of Technology (Project Number FSI‐S‐26‐9044); by the Technology Agency of the Czech Republic (TACR) (Grant FW06010042); by the Czech Science Foundation (Grant 22‐02794S to Marcela Buchtová and 25‐16166S to Pavel Pořízka) and by Brno University of Technology (Project Number CEITEC VUT/FSI‐J‐23‐8365).

## Disclosure

All authors have read and approved the final version of the manuscript. Pavel Hurník had full access to all of the data in this study and takes complete responsibility for the integrity of the data and the accuracy of the data analysis. This article is a revised and extended version of a preprint previously made available on SSRN [51].

## Conflicts of Interest

The authors declare no conflicts of interest.

## Supporting Information

Additional supporting information can be found online in the Supporting Information section.

## Supporting information


**Supporting Information** This article includes six supporting information tables (Tables S1–S6). Table S1 lists the samples analyzed by LIBS, including the decalcifying agent used, the map size, and the number of acquired spectra per sample. Tables S2–S6 list, for each decalcifying agent (HNO_3_, HCOOH, DC1, Microdecfast, and Löwy’s solution, respectively), the examined teeth together with their weight and size differences before and after decalcification and the total decalcification time.

## Data Availability

Materials described in the manuscript, including all relevant raw data, are included in the manuscript or freely available to researchers wishing to use them for noncommercial purposes upon request. LIBS data are available at Zenodo.org under https://zenodo.org/records/13919313.
