# Supplementary material for: Comparative Assessment of Five Decalcification Methods for Human Dental Tissue: An Ex Vivo Laboratory Study
Source: Int J Dent. 2026 Aug 18;2026:9951057. doi: 10.1155/ijod/9951057 (PMC13482768; doi:10.1155/ijod/9951057)
Supplement: Supplementary file 1 [file IJOD-2026-9951057-s001.docx]

Comparative Assessment of Five Decalcification Methods for Human Dental Tissue: An Ex Vivo Laboratory Study

Anna Faruzelová^a^*, Jana Vaculová^b,c^*, Lucie Vrlíková^a,e^, Jan Štembírek^d,e^, Jakub Buday^a,g^, Valeria Skopelidou^b,c^, Marcela Buchtová^e,f^, Pavel Hurník^b,c,e^†, Pavel Pořízka^a,g^, Jozef Kaiser^a,g^

a. *CEITEC Brno University of Technology, Purkyňova 656/123, 61200 Brno, Czech Republic*
b. *Institute of Molecular and Clinical Pathology and Medical Genetics, University Hospital Ostrava, 17. listopadu 1790/5, 708 52 Ostrava-Poruba, Czech Republic*
c. *Institute of Molecular and Clinical Pathology and Medical Genetics, Faculty of Medicine, University of Ostrava, Syllabova 19, 703 00 Ostrava, Czech Republic*
d. *Department of Oral and Maxillofacial Surgery, University Hospital Ostrava, 17. listopadu 1790/5, 708 52 Ostrava-Poruba, Czech Republic*
e. *Laboratory of Molecular Morphogenesis, Institute of Animal Physiology and Genetics, Czech Academy of Sciences, Veveří 967/97, 602 00 Brno, Czech Republic*
f. *Department of Experimental Biology, Masaryk University, Brno, Czech Republic*
g. *Faculty of Mechanical Engineering, Brno University of Technology, Technická 2896/2, 61669 Brno, Czech Republic*

* These authors contributed equally.

† Corresponding author: Pavel Hurník, pavel.hurnik@gmail.com

Table S1: List of samples analyzed by LIBS, their decalcifying agents, map sizes, and the number of acquired spectra.

| **decalcifying agent** | **sample** | **map size** | **spectra** |
| --- | --- | --- | --- |
| HNO_3_ | 1a | 580 × 560 | 324 800 |
|  | 1b | 601 × 510 | 306 510 |
|  | 10a | 422 × 278 | 117 316 |
|  | 10b | 481 × 280 | 134 680 |
| HCOOH | 6a | 521 × 365 | 190 165 |
|  | 6b | 636 × 390 | 248 040 |
|  | 10a | 485 × 305 | 147 925 |
|  | 10b | 421 × 275 | 115 775 |
| DC1 | 3DC | 486 × 225 | 162 810 |
|  | 4DC | 430 × 250 | 107 500 |
|  | 5DC | 330 × 270 | 89 100 |
|  | 6DC | 651 × 310 | 201 810 |
| Microdecfast | 3a | 531 × 330 | 175 230 |
|  | 3b | 491 × 350 | 171 850 |
|  | 10a | 461 × 296 | 136 459 |
|  | 10b | 500 × 355 | 177 500 |
| Löwy's solution | L1 | 461 × 260 | 119 860 |
|  | L4 | 521 × 375 | 195 375 |
| Non-decalcified (control) | 1 | 415 × 340 | 141 100 |

Table S2: List of examined teeth from HNO_3_ decalcification, their weight and size differences, and decalcification time.

| **sample** | **weight (g)** | | **size (cm)** | | **decalcification time (hours)** |
| --- | --- | --- | --- | --- | --- |
|  | **before decalcification** | **after decalcification** | **before decalcification** | **after decalcification** |  |
| N1 | 2.98 | 1.59 | 2.0 × 1.4 × 0.8 | 1.7 × 1.4 × 0.7 | 312 |
| N2 | 1.27 | 0.49 | 1.8 × 1.0 × 0.6 | 1.5 × 0.8 × 0.5 | 192 |
| N3 | 2.17 | 0.74 | 2.0 × 1.0 × 0.9 | 1.7 × 0.8 × 0.7 | 192 |
| N4 | 2.53 | 0.96 | 2.0 × 1.6 × 0.9 | 1.8 × 1.0 × 0.8 | 235 |
| N5 | 2.02 | 0.60 | 2.0 × 1.0 × 1.0 | 1.5 × 0.8 × 0.8 | 192 |
| N6 | 2.89 | 1.25 | 2.0 × 1.6 × 0.7 | 1.9 × 1.5 × 0.8 | 312 |
| N7 | 1.63 | 0.51 | 1.6 × 1.0 × 0.9 | 1.2 × 0.7 × 0.7 | 168 |
| N8 | 1.43 | 0.46 | 1.9 × 1.0 × 0.7 | 1.4 × 0.7 × 0.7 | 192 |
| N9 | 2.07 | 0.66 | 2.0 × 1.7 × 0.7 | 1.4 × 1.0 × 0.7 | 235 |
| N10 | 0.82 | 0.27 | 1.5 × 0.7 × 0.7 | 1.1 × 0.6 × 0.5 | 168 |

Table S3: List of examined teeth from HCOOH decalcification, their weight and size differences, and decalcification time.

| **sample** | **weight (g)** | | **size (cm)** | | **decalcification time (hours)** |
| --- | --- | --- | --- | --- | --- |
|  | **before decalcification** | **after decalcification** | **before decalcification** | **after decalcification** |  |
| F1 | 2.88 | 0.95 | 2.0 × 1.2 × 1.0 | 1.8 × 1.0 × 0.8 | 887.5 |
| F2 | 1.89 | 0.66 | 1.8 × 1.2 × 0.7 | 1.2 × 1.0 × 0.7 | 642.0 |
| F3 | 2.82 | 0.97 | 2.0 × 1.2 × 1.0 | 1.4 × 0.7 × 0.4 | 887.5 |
| F4 | 2.20 | 0.88 | 2.0 × 1.3 × 0.7 | 2.0 × 1.0 × 0.6 | 642.0 |
| F5 | 2.63 | 0.93 | 2.2 × 1.3 × 1.1 | 1.8 × 0.8 × 0.5 | 887.5 |
| F6 | 2.85 | 1.02 | 2.2 × 1.0 × 1.0 | 1.8 × 1.0 × 0.8 | 887.5 |
| F7 | 2.31 | 0.80 | 2.0 × 1.4 × 1.0 | 1.4 × 0.8 × 0.6 | 887.5 |
| F8 | 2.09 | 0.64 | 2.0 × 1.1 × 0.9 | 1.5 × 0.8 × 0.7 | 642.0 |
| F9 | 2.05 | 0.70 | 2.0 × 1.1 × 1.0 | 1.5 × 0.8 × 0.5 | 642.0 |
| F10 | 1.50 | 0.52 | 1.5 × 1.2 × 0.8 | 1.0 × 1.0 × 0.5 | 546.0 |

Table S4: List of examined teeth from DC1 decalcification, their weight and size differences, and decalcification time.

| **sample** | **weight (g)** | | **size (cm)** | | **decalcification time (months)** |
| --- | --- | --- | --- | --- | --- |
|  | **before decalcification** | **after decalcification** | **before decalcification** | **after decalcification** |  |
| D1 | 2.21 | 0.79 | 1.9 × 1.0 × 0.5 | 1.4 × 0.6 × 0.3 | 5 |
| D2 | 0.78 | 0.26 | 1.0 × 0.4 × 0.3 | 0.8 × 0.2 × 0.2 | 5 |
| D3 | 0.65 | 0.21 | 0.8 × 0.6 × 0.7 | 0.6 × 0.5 × 0.5 | 5 |
| D4 | 2.88 | 1.03 | 1.5 × 0.6 × 0.4 | 1.2 × 0.4 × 0.3 | 5 |

Table S5: List of examined teeth from Microdecfast agent decalcification, their weight and size differences, and decalcification time.

| **sample** | **weight (g)** | | **size (cm)** | | **decalcification time (hours)** |
| --- | --- | --- | --- | --- | --- |
|  | **before decalcification** | **after decalcification** | **before decalcification** | **after decalcification** |  |
| M1 | 2.21 | 0.70 | 1.5 × 1.0 × 0.8 | 1.3 × 0.9 × 0.7 | 642 |
| M2 | 2.33 | 0.78 | 1.6 × 0.9 × 0.7 | 1.5 × 0.7 × 0.5 | 642 |
| M3 | 1.04 | 0.43 | 1.6 × 0.7 × 0.7 | 1.4 × 0.7 × 0.7 | 146 |
| M4 | 2.02 | 0.68 | 1.8 × 1.0 × 0.7 | 1.3 × 0.7 × 0.5 | 642 |
| M5 | 1.63 | 0.65 | 1.5 × 1.0 × 0.9 | 1.2 × 1.0 × 0.6 | 480 |
| M6 | 1.81 | 0.56 | 1.5 × 1.0 × 0.8 | 1.4 × 0.7 × 0.6 | 481 |
| M7 | 2.03 | 0.77 | 1.4 × 1.0 × 0.9 | 1.0 × 1.0 × 0.7 | 482 |
| M8 | 3.03 | 0.96 | 2.2 × 1.0 × 1.0 | 1.9 × 0.9 × 0.9 | 642 |
| M9 | 1.21 | 0.55 | 1.9 × 0.8 × 0.7 | 1.7 × 0.7 × 0.5 | 480 |
| M10 | 1.70 | 0.57 | 1.9 × 0.9 × 0.8 | 1.5 × 0.7 × 0.6 | 642 |
| M11 | 2.00 | - | 2.0 × 1.0 × 0.8 | - | samples processed in a thermostat. disintegrated |
| M12 | 1.86 | - | 1.8 × 1.0 × 0.7 | - | samples processed in a thermostat. disintegrated |
| M13 | 1.86 | - | 1.6 × 0.8 × 0.7 | - | samples processed in a thermostat. disintegrated |
| M14 | 2.47 | - | 1.8 × 1.2 × 0.7 | - | samples processed in a thermostat. disintegrated |
| M15 | 1.61 | - | 1.9 × 1.1 × 0.5 | - | samples processed in a thermostat. disintegrated |

Table S6: List of examined teeth from Löwy’s solution decalcification, their weight and size differences, and decalcification time.

| **sample** | **weight (g)** | | **size (cm)** | | **decalcification time (months)** |
| --- | --- | --- | --- | --- | --- |
|  | **before decalcification** | **after decalcification** | **before decalcification** | **after decalcification** |  |
| L1 | 1.90 | 1.39 | 1.0 × 0.8 × 1.7 | 0.8 × 0.7 × 1.5 | 6 |
| L2 | 2.85 | - | 2.3 × 1.1 × 0.9 | - | did not decalcified |
| L3 | 2.21 | - | 2.0 × 1.1 × 0.7 | - | did not decalcified |
| L4 | 2.13 | 1.70 | 1.2 × 0.9 × 1.5 | 1.0 × 0.8 × 1.3 | 6 |
| L5 | 2.88 | - | 2.1 × 1.5 × 0.8 | - | did not decalcified |
| L6 | 2.30 | - | 1.8 × 1.0 × 0.7 | - | did not decalcified |
| L7 | 2.18 | - | 1.9 × 1.2 × 0.8 | - | did not decalcified |
| L8 | 2.75 | - | 2.1 × 1.2 × 0.7 | - | did not decalcified |
| L9 | 1.60 | 1.20 | 1.6 × 1.1 × 0.7 | 1.3 × 0.9 × 0.6 | 8 |
| L10 | 2.73 | 2.42 | 2.1 × 1.6 × 0.8 | 1.8 × 1.4 × 0.7 | 6 |
